# (*R*)-Desmolactone Is a Sex Pheromone or Sex Attractant for the Endangered Valley Elderberry Longhorn Beetle *Desmocerus californicus dimorphus* and Several Congeners (Cerambycidae: Lepturinae)

**DOI:** 10.1371/journal.pone.0115498

**Published:** 2014-12-18

**Authors:** Ann M. Ray, Richard A. Arnold, Ian Swift, Philip A. Schapker, Sean McCann, Christopher J. Marshall, J. Steven McElfresh, Jocelyn G. Millar

**Affiliations:** 1 Department of Biology, Xavier University, Cincinnati, Ohio, United States of America; 2 Entomological Consulting Services, Ltd., Pleasant Hill, California, United States of America; 3 California State Collection of Arthropods, Sacramento, California, United States of America; 4 Department of Integrative Biology, Oregon State University, Corvallis, Oregon, United States of America; 5 Department of Biological Sciences, Simon Fraser University, Burnaby, British Columbia, Canada; 6 Department of Entomology, University of California Riverside, Riverside, California, United States of America; Rutgers University, United States of America

## Abstract

We report here that (4*R*,9*Z*)-hexadec-9-en-4-olide [(*R*)*-*desmolactone] is a sex attractant or sex pheromone for multiple species and subspecies in the cerambycid genus *Desmocerus.* This compound was previously identified as a female-produced sex attractant pheromone of *Desmocerus californicus californicus*. Headspace volatiles from female *Desmocerus aureipennis aureipennis* contained (*R*)*-*desmolactone, and the antennae of adult males of two species responded strongly to synthetic (*R*)*-*desmolactone in coupled gas chromatography-electroantennogram analyses. In field bioassays in California, Oregon, and British Columbia, traps baited with synthetic (*R*)*-*desmolactone captured males of several *Desmocerus* species and subspecies. Only male beetles were captured, indicating that this compound acts as a sex-specific attractant, rather than as a signal for aggregation. In targeted field bioassays, males of the US federally threatened subspecies *Desmocerus californicus dimorphus* responded to the synthetic attractant in a dose dependent manner. Our results represent the first example of a “generic” sex pheromone used by multiple species in the subfamily Lepturinae, and demonstrate that pheromone-baited traps may be a sensitive and efficient method of monitoring the threatened species *Desmocerus californicus dimorphus*, commonly known as the valley elderberry longhorn beetle.

## Introduction

Volatile pheromones are widely used for detection, monitoring, and in some cases, control of insect pests [1], [2], and a substantial amount of research has been devoted to the development of methods to exploit pheromones for effective pest management. These efforts have included the development of pheromone-based monitoring methods for invasive insect pests such as the gypsy moth *Lymantria dispar* (L.) and the Japanese beetle *Popillia japonica* Newman [3], [4]. Such methods are particularly useful for detection of invasive species because of their sensitivity and selectivity, i.e., pheromone-baited traps can be used to detect very low density populations, such as occur when an invasive pest is first introduced, with minimal cross-attraction of non-target species [5]. In addition, pheromone-based monitoring methods are generally far simpler, far more effective, and less costly and labor intensive than visual searches for a target pest or signs of infestation [6].

The same characteristics of sensitivity and selectivity should make pheromone-based sampling methods an ideal means of detecting and sampling rare, threatened, or endangered species. Despite these obvious advantages, pheromone-based methods have been exploited for these purposes in only a very few cases (e.g., the moth *Actias* ( =  *Graellsia*) *isabellae* (Graëlls) [7], [8]; wood cockroaches as food for endangered bird species [9]; the scarab beetle *Osmoderma eremita* Scopoli and the click beetle *Elater ferrugineus* L. [10]) in part because of the high cost of identifying and developing pheromones as a means to study populations of rare or endangered species [10].

The valley elderberry longhorn beetle (VELB), *Desmocerus californicus dimorphus* Fisher (Coleoptera: Cerambycidae), was listed as threatened by the US Fish and Wildlife Service (USFWS) in 1980 [11]. Since that time, *D. californicus dimorphus* has been the subject of extensive research, including studies of the effects of invasive ants [12], dust [13], habitat fragmentation [14], and outcomes of various mitigation plans on beetle populations [13], [15]–[18]. In response to a petition by a private legal foundation, the USFWS proposed to remove VELB from the Federal List of Endangered and Threatened Wildlife in 2012, using population density and distribution data summarized in a 5-year review of the status of the beetle as the basis for the proposed delisting [19], [20]. These data were collected by laborious and relatively ineffective visual surveys for adult beetles and for beetle emergence holes (e.g., [14], [20]). Panelists who reviewed the proposal to delist VELB noted a need to develop more sensitive and less labor-intensive survey methods, such as a pheromone or attractant lure that could be used to reliably detect beetles at low population densities [21].

Over the past decade, extensive progress has been made in the identification of volatile sex or aggregation pheromones for cerambycid beetles, with pheromones or suspected pheromones now known for several hundred species in the subfamilies Cerambycinae, Lamiinae, Prioninae, Spondylidinae, and Lepturinae (e.g., [22]–[27]). Overall, these results have demonstrated that, as with many other insects, volatile pheromones play a crucial role in mate location in this family. Recent studies also have demonstrated the utility of pheromones as a sensitive tool for surveying cerambycid species in a variety of habitats (e.g., [22], [28]), with extensive data sets generated on seasonal phenology, geographic range, and distribution for a number of species (e.g., [29], [22], [30]).

We recently identified the female-produced sex pheromone of *Desmocerus californicus californicus* Horn as a single compound, (4*R*,9*Z*)-hexadec-9-en-4-olide, given the common name (*R*)-desmolactone. This pheromone was attractive to male *D. californicus californicus* in field trials conducted in southern California [31]. Because pheromone structures are highly conserved within and among related cerambycid species (e.g., [22], [32]), we hypothesized that female VELB also might use (*R*)-desmolactone as a sex pheromone. If this were the case, the pheromone could be used as a tool to obtain more accurate estimates of population sizes and distributions than can be obtained from visual surveys for adults or for emergence holes. We also reasoned that (*R*)-desmolactone may be a “generic” pheromone for other species and subspecies of *Desmocerus.*

Thus, the objectives of the present study were: 1) to determine whether (*R*)-desmolactone was an attractant for VELB, 2) to test (*R*)-desmolactone and its enantiomer as an attractant or sex pheromone for other North American *Desmocerus* species and subspecies, 3) where possible, to analyze headspace volatiles and conduct coupled gas chromatography-electroantennogram analyses to confirm that female beetles produce (*R*)-desmolactone. We also tested the antennal responses of *Desmocerus* species males to synthetic (*R*)- and (*S*)-desmolactone.

### Biology and Distribution of Species and Subspecies

**Figure 1:**
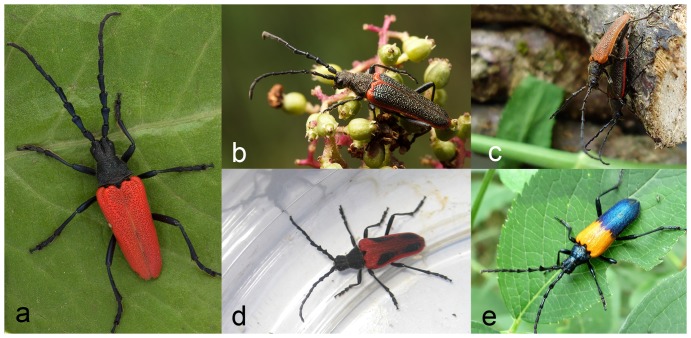
Photos of the species and subspecies of *Desmocerus* included in the present manuscript. A: *D. a. aureipennis;* B: *D. a cribripennis*; C: *D. a. lacustris*; D. *D. c. dimorphus* (VELB); E. *D. palliatus.* (Photo of *D. palliates* by Paul Bedell).

The genus *Desmocerus* Dejean in the cerambycid subfamily Lepturinae is endemic to North America, and contains three species and seven recognized subspecies [33], [34]. Both subspecies of *Desmocerus californicus* are native to California, but VELB is restricted to the Sacramento and San Joaquin River valleys [33], [35]. The four subspecies of *D. aureipennis* are found in mountain and coastal regions west of the Rocky Mountains in California, Oregon, and Washington in the United States, and British Columbia in Canada. The nominate subspecies, *D. a. aureipennis* Chevrolat, is distributed from the Sierra Nevada Mountains (California) north to Vancouver Island (British Columbia, Canada) [34], *D. aureipennis cribripennis* Horn is found along the Pacific Coast from central California to the Queen Charlotte Islands in Canada [PAS, pers, obs.], [34], *D. aureipennis lacustris* Linsley and Chemsak is endemic to the mountains of western Oregon [34], and *D. aureipennis piperi* Webb is found in the northern Rocky Mountains [34], [36] (the latter subspecies is not included in the present manuscript). For simplicity, we follow the taxonomy of *D. aureipennis* as described by Linsley and Chemsak [34]. However, morphological characters are often not sufficient to separate individuals of different Pacific Northwest populations of *D. aureipennis* (e.g., [34]), and nomenclatural changes are anticipated [PAS, pers. obs.]. *Desmocerus palliatus* (Forster) occurs throughout eastern North America from Ontario, Canada to Alabama and northern Georgia, and west to Kansas in the United States [34] (see Fig. 1 for photos of species and subspecies included in the present manuscript).

The biology of all *Desmocerus* species and subspecies is similar [34]. Members of the genus are unusual among lepturines because their larvae develop in stems and roots of living elderberry (*Sambucus* spp.: Adoxaceae) rather than in decaying wood [34], [37]–[39]. Adults emerge from March to August (depending on elevation and latitude), and feed on the leaves and flowers of the host plant [34], [40]. Adults mate on the host, and females lay eggs in bark crevices [41]. Adults are seldom encountered in the field [13], [42], and specimens of most taxa are fairly uncommon in entomology collections [31].

## Methods and Materials

### Field Bioassays

Field bioassays were conducted in California, Missouri, Oregon (USA), and British Columbia (Canada) during May - August 2012 and April - June 2013 (see Table 1 for site information). We tested responses of adult VELB (California), *D. aureipennis* subspecies (Oregon and British Columbia), and *D. palliatus* (Missouri) to the enantiomers and/or the racemate of desmolactone (Table 2). Funnel traps as described in Ray et al. [31] were used at all sites. Trap funnels were treated with a fluoropolymer suspension (Fluon, Northern Products Inc., Woonsocket, RI, USA) to render the surfaces slippery and improve capture of beetles [43]. Traps were positioned ∼10 m apart in approximately linear transects and were suspended from branches of *Sambucus* shrubs or from branches of other tree species near patches of *Sambucus* (see Table 1 for host species). Pheromone lures consisted of clear low-density polyethylene sachets (“Zipper” seal sample bags, 2 in ×3 in, 0.002 in wall thickness, #01-816- 1A; Fisher Scientific, Pittsburgh, PA, USA), which were loaded with solutions of (*R*)- or (*S*)-desmolactone (10 mg in 990 µl of ethanol), 20 mg of the racemic mixture (in 980 µl ethanol), or 1 ml ethanol only as a control. (*R*)- and (*S)*-desmolactones [(4*R*,9*Z*)- and (4*S,*9Z)- hexadec-9-en-4-olides] were synthesized as described in Ray et al. [31]. Lures were suspended with wire from the side of the funnel (see Table 2 for treatments and replicates at each site).

**Table 1:** Location data, voucher numbers, *Desmocerus* species targeted, and host plant species for bioassays testing attraction to synthetic pheromones.

Site	City, State/Province	GPS coordinates	Species	Host plant	Voucher numbers of specimens
Mt. Ashland	Ashland, OR	42.081, −122.705	*D. aureipennis aureipennis*	*Sambucus nigra* ssp. *cerulea* (Rafinesque) R. Bolli	OSAC 000556818-00055683
Colony Farm Park	Coquitlam, BC	49.238, −122.797	*D. aureipennis cribripennis*	*S. racemosa* ssp. *racemosa* L.	OSAC 000712241-000712270; UCR ENT 438041-438045
Mill Creek	Logsden, OR	44.763, −123.787	*D. aureipennis lacustris*	*S. racemosa* ssp. *racemosa*	OSAC 000556515-000556519
Sacramento River National Wildlife Refuge	Willows, CA	39.627, −121.999	*D. californicus dimorphus*	*S. nigra* ssp. *cerulea*	OSAC 000556491-000556493; UCR ENT 205324-205326
Squaw Creek National Wildlife Refuge	Mound City, MO	40.092, −95.267	*D. palliatus*	*S. nigra* ssp. *canadensis* (L.) R. Bolli	OSAC 000556523-000556523

Taxonomy of host plants follows [52]. GPS coordinates indicate the position of the first trap of the first replicate. Voucher specimens of all species and subspecies were submitted to the Oregon State Arthropod Collection (OSAC, Corvallis, OR, USA). Additionally, vouchers of *D. aureipennis cribripennis* and *D. californicus dimorphus* were submitted to the Entomology Research Museum at the University of California, Riverside (UCR ENT).

**Table 2:** Summary of treatments and results for bioassays testing attraction of three *Desmocerus* species to synthetic pheromones.

Species	Study region	Dates	# Blocks/site	# Trap checks	Friedman's *Q* a	Bioassay treatments	Mean ±SE # captured
*D. a. aureipennis*	OR (Ashland)	3–10 August 2012	2	1	NS	(*R*)-desmolactone	11.0±1.0a
						Racemic desmolactone	2.5±1.5a
						Control	0a
*D. a. cribripennis*	BC	6 May–3 June 2013	2	4	*Q*_3,8_ = 15.13*	(*R*)-desmolactone	29.0±21.73a
						(*S*)-desmolactone	0.5±0.38b
						Racemic desmolactone	21.0±10.0a
						Control	0.13±0.13b
*D. a. lacustris*	OR (Logsden)	20 May–14 June 2012	2	3	NS	(*R*)-desmolactone	1.66±0.33a
						Racemic desmolactone	0b
						Control	0b
*D. c. dimorphus*	CA	2–26 April 2013	3	6	*Q*_3,17_ = 61.72**	(*R*)-desmolactone	1.94±0.39a
						(*S*)-desmolactone	0b
						Racemic desmolactone	0.6±0.06b
						Control	0b
*D. palliatus*	MO	15 May–20 June 2012	1	6	NS	(*R*)-desmolactone	3.5±2.5a
						(*S*)-desmolactone	0a
						Racemic desmolactone	0a
						Control	0a

Data for *D. a. cribripennis* (BC) were square root transformed because number of beetles captured varied considerably across dates. Untransformed mean ±SE are listed here. Means within species with the same letters are not different at *P*≤0.05 (REGWQ means-separation test, [44]).

a Asterisks indicate a significant value of *Q*: *P<0.0005, **P<0.0001; NS denotes that treatment means were not significantly different at *P*≤0.05.

Traps were checked for beetles every 2-7 d (Table 2). Lures were replaced after 7 d and traps were shifted along transects to control for location effects. Captured beetles were sexed using the sexually dimorphic characters of antennal length, width of the elytra, color of the elytra (in sexually dichromatic species), and shape of the terminal abdominal sternite [34].

Differences between treatments in the number of beetles captured per trap per trapping period were tested with the nonparametric Friedman's test (blocked by date and replicate; PROC FREQ with CMH option; [44]) because assumptions of analysis of variance were violated by heteroscedasticity [45]. We excluded from analyses any dates when no beetles were captured. Data from bioassays conducted in British Columbia were square root transformed due to the great range in number of beetles captured across dates (see Results). Differences between pairs of means were tested with the REGWQ means-separation test to control maximum experimentwise error rates [44].

All work with *D. californicus dimorphus* was carried out under USFWS recovery permit # 797233 (to RAA), and work at the Sacramento River National Wildlife Refuge was carried out under USFWS SUP#81627-13-0010 (to RAA). Work at the Squaw Creek National Wildlife Refuge was carried out under USFWS SUP #33560-2012-0012 (to AMR). Work at the Logsden, OR field site was conducted on private land, and permission was granted by the property owner, David Nafzinger (to PAS). Work at the Ashland, OR field site was conducted on private land, and permission was granted by Woodie Tesh at Mt. Ashland Ski Area (to PAS). Work at Colony Farm in BC was conducted under a Metro Vancouver Parks Board permit issued to SM.

### Source of Beetles for Headspace Analyses and/or Coupled Gas Chromatography-Electroantennogram Detection (GC-EAD)

Adults of *D. a. aureipennis* were collected from leaves of *S. nigra* ssp. *cerulea* on 2 August 2012 at the Ashland, OR field site (Table 1; see above for permission). One female and one male were collected *in copula*, while the mating status of other individuals was unknown. Adult males of *D. a. lacustris* were collected in traps baited with (*R*)-desmolactone at the Logsden, OR field site (Table 1; see above for permission).

Pupae and teneral adults of *D. palliatus* were collected 4–6 May 2012 from healthy *S. nigra* ssp. *canadensis* plants growing at the Squaw Creek National Wildlife Refuge near Mound City in northwest Missouri (see Table 1; see above for permit information). Pupae and teneral adults were cut from pupal chambers in the roots of infested plants. Individual beetles were placed into tissue paper tubes (∼6 cm long, ∼0.5 cm in diameter), and the tube was misted with water. Paper tubes were placed in individual glass vials, and shipped to the University of California, Riverside for chemical analysis. All adults had emerged upon arrival in California, but only males survived for analysis.

Shipments of live beetles followed the protocols required by USDA permit P526P-09-01886 (to JGM).

### Collection of Volatiles

Volatiles were collected from male and female *D. a. aureipennis* in a greenhouse under ambient conditions between 9 and 13 August 2012. Headspace odors were collected from either two males held together or two single females held in separate glass chambers made from 500 ml canning jars with Teflon lids. The results of field bioassays (see below) suggested that females would produce attractants, and that males would not. Thus, we collected headspace from individual females to increase the likelihood of obtaining replicated samples from the limited numbers of specimens available to us. Stems of *S. nigra* ssp. *cerulea* (with inflorescences and leaves) were provided as a food source and replaced every 1–2 d, as needed. Airflow of ca.1 l/min was provided by a flow meter-controlled vacuum source with charcoal-filtered (6-14 mesh, Fisher Scientific, Pittsburgh, PA, USA) room air being pulled through the chamber. Collectors consisted of 6 mm outer diameter glass tubes with ca. 1.5–2 cm long beds of thermally desorbed 50–200 mesh activated charcoal (Fisher Scientific, Pittsburgh, PA, USA) held in place by Soxhlet-extracted (pentane, then ethyl acetate) glass wool plugs. Collectors were connected to the vacuum source and the chamber using ¼ in to ¼ in Swagelok unions, with Teflon ferrules (Swagelok, San Diego Valve and Fitting Co., San Diego CA, USA). Volatiles were collected for 4 d, after which the collectors were eluted with 3 rinses of dichloromethane to a final volume of ca. 250 µl. Extracts were stored at ∼−20°C until needed.

### Analyses of Extracts

Coupled GC-EAD analyses were performed with DB-17 and DB-Wax columns (both 30 m × 0.25 mm i.d., 0.25 µm film; J&W Scientific, Folsom, CA, USA). Helium was used as carrier and makeup gas. The GC oven was programmed from 100°C for 1 min, 15°C per min to 275°C for DB-17 and 250°C for DB-Wax, hold for 40 min. Extracts (1 µl aliquots) were analyzed in splitless mode. The effluent from the columns was split equally between the GC detector and the EAD. The portion directed to the EAD was diluted in a humidified air stream that was directed over the antennal preparation, which consisted of the excised terminal 4–5 antennal segments of a male beetle, with the distal tip excised with a razor blade, placed between two saline-filled glass capillary electrodes (7.5 g NaCl, 0.21 g CaCl_2_, 0.35 g KCl, and 0.20 g NaHCO_3_ in 1 l Milli-Q purified water). The glass electrodes were fitted with 0.2 mm diam. gold wires that connected to a custom built EAD amplifier, with the amplifier gain at 100X amplification. A single antennal preparation was used for as many as ten runs.

We tested the responses of the antenna of a single male *D. a. aureipennis* to extracts of headspace volatiles collected from females (replicated three times), to headspace volatiles collected from males (replicated twice), and to synthetic (*R*)-desmolactone (one replicate). We tested the responses of an antenna from each of two male *D. a. lacustris* to synthetic (*R*)- and (*S*)-desmolactone (maximum of two GC-EAD runs per male, but multiple injections of standards during each run). Responses of an antenna from each of three individual male *D. palliatus* to synthetic (*R*)- and (*S*)-desmolactone were also tested (one GC-EAD run per individual male, but multiple injections of standards during each run). Extracts of headspace volatiles from female *D. a. lacustris* and *D. palliatus* were not available (see above). The differences in the number of GC-EAD analyses between species were a direct result of the limited number of individual beetles available (see above).

Aeration samples also were analyzed by gas chromatography-mass spectrometry with an Agilent 6890N GC interfaced to a 5975C mass selective detector (Agilent, Santa Clara CA, USA). The GC was equipped with an HP5-MS column (30 m×0.25 mm i.d., 0.25 µm film; Agilent, Santa Clara CA, USA), programmed from 40°C/1 min, 10°C per min to 280°C. Samples were injected in splitless mode.

A sample from a female *D. auripennis auripennis* that contained desmolactone was reanalyzed on a Betadex B chiral stationary phase GC column (30 m×0.25 mm i.d., 0.25 µm film; J&W Scientific, Folsom CA), programmed from 185°C for 50 min, then 10°C per min to 220° for 40 min. (*R*)- and (*S*)-desmolactone standards were run under the same conditions, with retention times of 39.17 and 40.04 min respectively.

### Dose Response Study

We tested the responses of adult male VELB to different doses of synthetic (*R*)-desmolactone during field bioassays at the Sacramento River National Wildlife Refuge from 27 March–16 May 2014 (see S1 Figure, S1 Table). Traps and lures were as described above, but traps were separated by ∼50 m to minimize interference between adjacent traps. We tested responses of adult male VELB to traps baited with 1, 3.3, 10, or 33 mg doses of (*R*)-desmolactone in 0.5 ml isopropyl alcohol (solvent), versus a solvent control. Each block contained five traps, each trap was baited with one of the five treatments, and six total blocks were deployed in three separate conservation units within the refuge (see S1 Table). Traps were checked every 7–10 d for the duration of the bioassay, resulting in six trap checks. Traps were shifted one position along the transect every 7 d to control for location effects, and lures were replaced every 14 d. The dose response study was conducted under the USFWS permits listed above (to RAA). Statistical analyses were as described above, and trapping periods when no beetles were captured were excluded from analysis.

## Results

### Field Bioassays

In bioassays in California testing attraction to synthetic pheromone, traps baited with (*R*)*-*desmolactone captured 33 male VELB, whereas traps baited with racemic desmolactone captured a single male (Table 2). No beetles were captured in traps baited with the (*S*)-enantiomer or in control traps. As many as seven male beetles were caught in a single trap during one five-day trapping period. We did not capture any female beetles, nor did we capture any other insects in consistent or significant numbers.

During bioassays in British Columbia, traps baited with (*R*)-desmolactone captured 232 males of *D. aureipennis cribripennis*, whereas traps baited with the racemate captured 168 males, and traps baited with the (*S*)-desmolactone and control treatments caught one male each (Table 2). A maximum of 180 male beetles were captured in a single trap baited with (*R*)-desmolactone, and a maximum of 80 male beetles were captured in a trap baited with racemic desmolactone during one seven-day trapping period.

In Logsden, Oregon, traps baited with (*R*)-desmolactone captured five male *D. a. lacustris*, whereas traps baited with racemic desmolactone and the solvent control captured no beetles (Table 2). Bioassays in Ashland, Oregon captured 22 male *D. a. aureipennis* in traps baited with (*R*)-desmolactone, with a maximum of 12 beetles in a single trap during one seven-day trapping period. Five male beetles were captured in traps baited with racemic desmolactone, with a maximum of four beetles in a single trap during a trapping period. No beetles were captured in control traps at the Ashland site (Table 2).

Bioassays in Missouri testing attraction of *D. palliatus* to (*R*)- and (*S*)-desmolactones captured seven male beetles in traps baited with (*R*)-desmolactone. A maximum of six individuals were captured in a single trap during a single seven-day trapping period, and no beetles were caught in traps baited with (*S*)-desmolactone or in control traps (Table 2). However, treatment means were not significantly different.

### Analysis of Headspace Extracts

**Figure 2:**
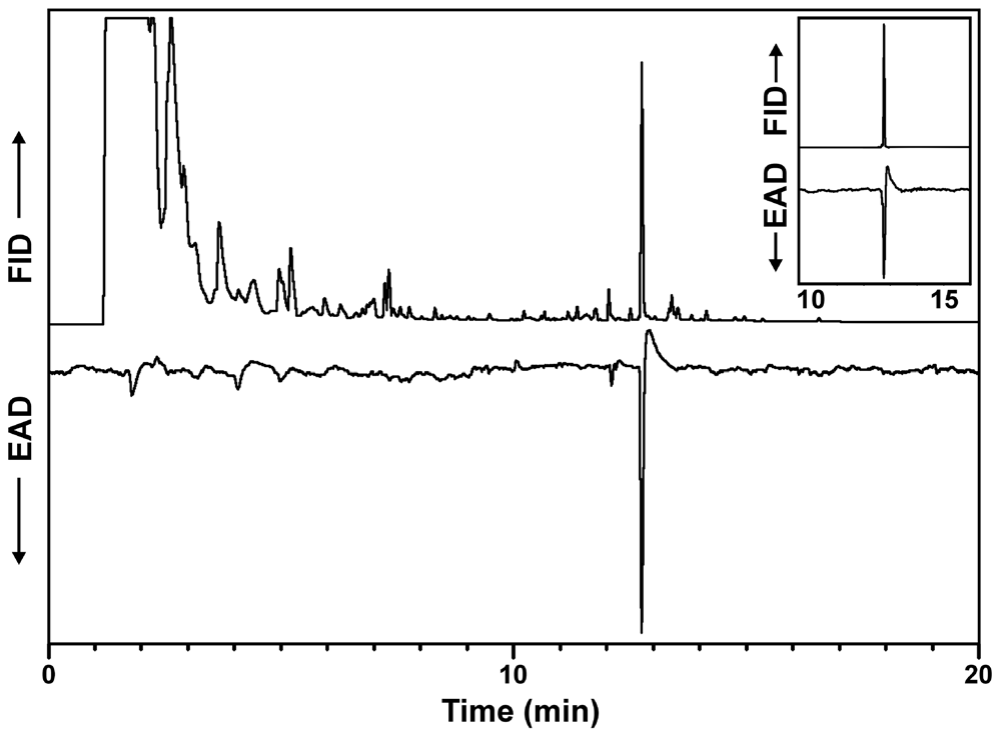
Representative coupled gas chromatography-electroantennogram analysis of an extract of headspace odors from a female *Desmocerus a. aureipennis.* Upper trace is the chromatogram, lower inverted trace is the electroantennogram signal from the antenna of a male beetle. Insert shows the GC and antennal responses to an authentic standard of (*R*)*-*desmolactone. DB-5 column, 40°C/1 min, then 10°C/min to 275°C, hold 40 min.

Analysis of headspace volatiles collected from adult *D. a. aureipennis* revealed a sex-specific peak in extracts from females that elicited strong responses from the antenna of a male beetle in GC-EAD analyses (Fig. 2). Analysis by coupled GC-MS confirmed that the mass spectrum and GC retention time of the active compound matched those of an authentic standard, and further analysis on a chiral stationary phase column demonstrated that the extract contained pure (*R*)-desmolactone. The extracts of headspace volatiles collected from male beetles did not contain desmolactone, nor any other compounds that elicited responses from antennae in GC-EAD analyses.

**Figure 3:**
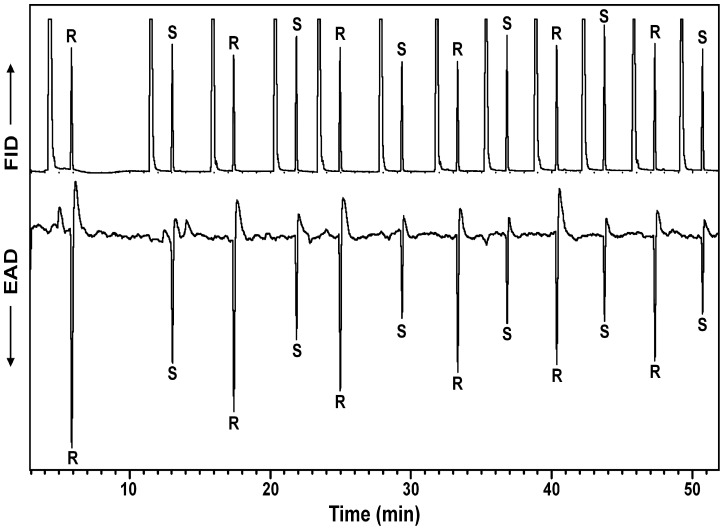
Representative coupled gas chromatography-electroantennogram analysis of repeated, alternate injections of synthetic (*R*)*-* and (*S*)-desmolactone using an antenna from a male *Desmocerus a. lacustris*. Upper trace is the chromatogram, lower inverted trace is the electroantennogram signal from the beetle antenna. DB-5 column, 40°C/1 min, then 10°C/min to 275°C, hold 40 min. Large peaks interspersed between the (*R*)*-* and (*S*)-desmolactone peaks on the GC trace are from the solvent used in the injections.

When challenged with synthetic (*R*)- and (*S*)-desmolactone, the antenna of a male *D. a. lacustris* responded to both enantiomers, with the response to the latter enantiomer being substantially weaker than that to the (*R*)-enantiomer (Fig. 3). The responses of the antennae of both male beetles that were available to test were consistent within each replicate.

**Figure 4:**
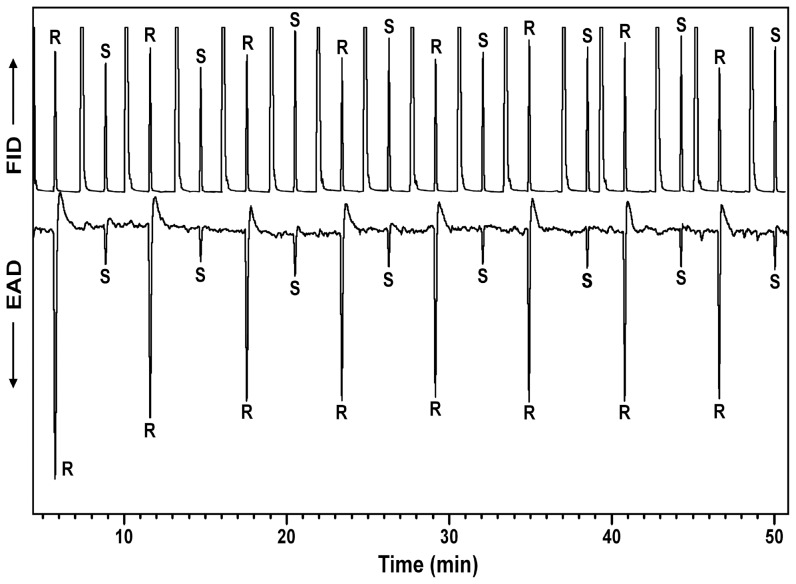
Representative coupled gas chromatography-electroantennogram analysis of repeated, alternate injections of synthetic (*R*)*-* and (*S*)- desmolactone using an antenna from a male *Desmocerus palliatus*. Upper trace is the chromatogram, lower inverted trace is the electroantennogram signal from the beetle antenna. DB-5 column, 40°C/1 min, then 10°C/min to 275°C, hold 40 min. Large peaks interspersed between the (*R*)*-* and (*S*)-desmolactone peaks on the GC trace are from the solvent used in the injections.

Antennae of male *D. palliatus* responded strongly to (*R*)-desmolactone, with a weaker response to (*S*)-desmolactone (Fig. 4). It was not possible to collect headspace volatiles from or conduct GC-EAD analyses with VELB because of permit prohibitions on handling or working with the beetles in the laboratory.

### Dose response study

**Figure 5:**
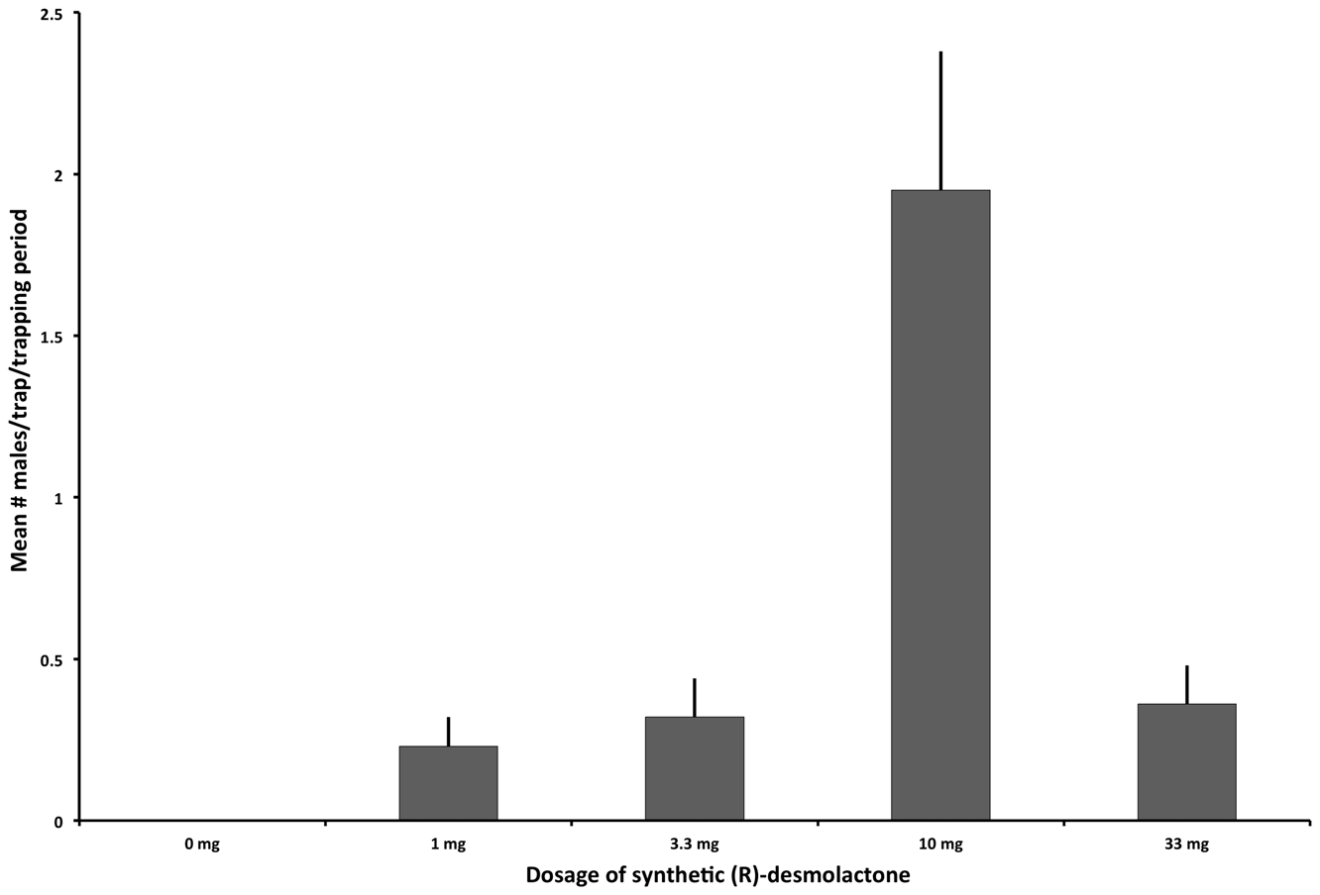
Captures of male VELB in traps baited with different doses of synthetic (*R*)-desmolactone (number of beetles captured in 10 mg treatment = 43, Friedman's *Q* = 27.31). Means with the same letters are not different at *P*≤0.0001 (REGWQ means-separation test [44]).

In bioassays to determine the optimal dose of pheromone for attracting VELB males, traps baited with different doses of synthetic (*R*)-desmolactone captured a total of 63 male VELB, whereas control traps caught no beetles. Beetles were captured at two of the three conservation units where traps were deployed. Treatments differed significantly in the number of beetles captured in traps. Traps baited with 10 mg dose captured significantly more beetles than traps baited with any other dose (number of beetles captured in 10 mg treatment = 43, Friedman's *Q* = 27.31, *P*<0.0001). Traps baited with the 10 mg doses captured a mean of 1.94±0.43 (SE) beetles per trapping period. Treatment means for the three other doses were not significantly different from solvent controls (Fig. 5).

## Discussion

In field bioassays in California, Oregon, Missouri, and British Columbia, traps baited with synthetic (*R*)-desmolactone or racemic desmolactone captured males of several *Desmocerus* species and subspecies. During field bioassays at the Sacramento River National Wildlife Refuge in 2013, we captured significantly more male VELB in traps baited with (*R*)-desmolactone, than in traps baited with the racemic desmolactone. This result suggests that the presence of the (*S*)-enantiomer at least partially inhibited the responses of males, as has been observed in tests of racemic versus enantiomerically pure pheromones of other cerambycid species (e.g., [46]).

Among the subspecies of *D. aureipennis*, we captured 400 males of *D. a. cribripennis* in traps baited with (*R*)-desmolactone and the racemate, four individuals in traps baited with (*S*)-desmolactone, and only one individual in the control. This result shows that male *D. a. cribripennis* are strongly attracted to the (*R*)-enantiomer, and the (*S*)-enantiomer does not entirely inhibit the response of males in this subspecies. During bioassays in Oregon, only small numbers of males were captured during bioassays, and low statistical power obscured any differences between treatments. Nevertheless, most of the *D. a. aureipennis* (Ashland) and *D. a. lacustris* (Logsden) males were captured in traps baited with (*R*)-desmolactone, suggesting that this compound has at least some level of field activity. We also captured few individuals of *D. palliatus* during bioassays in Missouri, and treatment means were not significantly different. Nevertheless, all of the beetles were captured in traps baited with (*R*)-desmolactone.

Only male *Desmocerus* were captured in all field trials, indicating that (*R*)-desmolactone is a sex attractant rather than an aggregation pheromone. Female-produced sex pheromones have been identified in one other lepturine species (*Ortholeptura valida,* [27]), and in several genera of the cerambycid subfamily Prioninae (e.g., *Prionus* [29] and *Tragosoma* [47]). In contrast, only male-produced pheromones that attract both sexes have been found to date in the cerambycid subfamilies Cerambycinae, Lamiinae, and Spondylidinae (reviewed by [22]). It is likely too early to draw conclusions regarding patterns in use of volatile pheromones in lepturines, because pheromones have been identified from species in only two of the 216 lepturine genera [48]. Moreover, among lepturines, both *Desmocerus* and *Ortholeptura* have unusual life histories, with larvae developing in living or recently dead hosts, rather than decomposed hosts [34]. As such, the adaptive significance and taxonomic distribution of volatile pheromone production in the Lepturinae remains to be determined.

Through analysis of headspace volatiles, we confirmed that *D. a. aureipennis* females produce (*R*)-desmolactone. We were unable to obtain females of the other species and subspecies to verify that they also produced this compound. Nevertheless, the strong and specific responses elicited from antennae of male *D. palliatus* to synthetic (*R*)-desmolactone suggest that this compound is at least one component of their sex pheromone. Due to limitations on our collecting permit, we were unable to use VELB of either sex for headspace or coupled GC-EAD analyses. Nevertheless, the data from *D. a. aureipennis* and *D. c. californicus* suggest that females of VELB are almost certain to produce (*R*)-desmolactone, a hypothesis supported by attraction of male VELB to traps baited with (*R*)-desmolactone.

Our results suggest that pheromone structures may be conserved within closely related lepturine species, as has been found with other cerambycid subfamilies [22]. We previously hypothesized that (*R*)-desmolactone might be a species-specific, rather than a generic, attractant because preliminary bioassays during summer 2011 did not capture individuals of *D. palliatus* or any of the subspecies of *D. aureipennis* [31]. However, the present more comprehensive study provides evidence that (*R*)-desmolactone is likely to be a generic attractant for males of all species in the genus *Desmocerus*. Furthermore, our results demonstrate that (*R*)-desmolactone alone is sufficient to attract male beetles to traps, suggesting that host plant volatiles are not critical for attraction of these species. Nevertheless, host plant volatiles may enhance attraction of beetles to traps baited with (*R*)-desmolactone, and, this may warrant further research.

In 2012, the USFWS reviewed the status of VELB populations and concluded that the beetle should be delisted, based on survey data suggesting that VELB no longer met the criteria for a threatened species [20]. However, the USFWS based the decision to delist on “minimal surveys” to assess geographic distribution of VELB populations, and admitted that there were “data uncertainties” as to whether sufficient habitat would remain for VELB if federal protection was eliminated [20]. As a result of a review of survey data, the USFWS delayed and recently has withdrawn the proposal to delist VELB [49]. Thus, there will likely be continued interest in exploration of more accurate methods for surveying and monitoring VELB populations. The current method for surveying for VELB consists of visual inspection of trees for adults or identification of emergence holes, the latter being prone to false positives and false negatives because beetles may emerge from roots below the soil line, emergence holes may be obscured over time, or holes made by other insects, such as woodboring bees, may be mistakenly attributed to VELB (e.g., [42], [50]).

The results from the research described here have important implications for preservation and restoration of habitat for VELB, because we have shown that pheromone-baited traps can provide a sensitive and efficient method of detecting VELB populations. For example, we captured 34 male VELB in 2013, a total which exceeds the total number of specimens known when the subspecies was originally recognized as threatened [20]. During the subsequent dose response survey in 2014, we captured an additional 63 males, showing that traps baited with (*R*)-desmolactone can be used to detect and document the geographic range of this subspecies. Traps baited with (*R*)-desmolactone exclusively attract males, and so females remain in the population, available for mating and oviposition. Furthermore, our current traps are designed to capture beetles alive (e.g., [31]), and careful placement and frequent monitoring of traps will minimize mortality and lessen potential effects of trapping on populations. The relatively low volatility of the pheromone suggests that lures will likely remain attractive for several weeks, and possibly for the entire flight season of VELB, so that replacement of the lures may not be necessary.

Traps baited with synthetic (*R*)-desmolactone are probably the most sensitive and effective method developed to date to assess presence of VELB. Protected conservation lands include extensive areas of habitat restored to benefit VELB (e.g., [14], [17]). However, because current survey methods for VELB are unreliable (see above), it is often impossible to know whether the site has been recolonized by VELB. Traps baited with (*R*)-desmolactone could be used for this purpose, and as such, could become a valuable tool for landowners and resource managers. In fact, our 2014 field bioassays at the Sacramento River National Wildlife Refuge were conducted at three conservation units where habitat restoration was initiated ∼15 years ago, including a riparian woodland, an elderberry savanna, and a mixed riparian scrub and woodland area (see S1 Figure, S1 Table). The fact that we captured beetles at two of three restored refuge sites demonstrates that the habitat restoration efforts have been effective at reestablishing or maintaining VELB populations.

In contrast, we did not capture any VELB at a third conservation unit, McIntosh Landing South, although traps were deployed in an area with abundant elderberry shrubs. Furthermore, no above-ground emergence holes were observed in any of the elderberry plants at this site. This result suggests that appropriate habitat is not necessarily an indication that VELB are present or have recolonized an area following extirpation and habitat restoration. Current federal guidelines require that habitat restoration activities to benefit VELB be monitored for a minimum of 10 years following initial restoration [51]. However, these guidelines do not require demonstration that VELB has indeed successfully recolonized a site. The results presented here suggest that the USFWS could revise its VELB Conservation Guidelines [51] to include the use of pheromone-baited traps to demonstrate successful recolonization by VELB at restoration sites, conservation banks that provide VELB credits, or other sites that are protected to provide habitat for the beetle.

## Supporting Information

S1 Figure **Map showing locations of three conservation units within the Sacramento River National Wildlife Refuge (Glenn Co., CA USA) where dose response studies were conducted for VELB.**
(TIFF)

S1 Table **Location data and descriptions of habitat for the three conservation units within the Sacramento River National Wildlife Refuge where dose response studies were conducted for VELB.** GPS coordinates indicate the position of the first trap in each replicate.
(DOC)
