# Supplementary material for: (R)-Desmolactone Is a Sex Pheromone or Sex Attractant for the Endangered Valley Elderberry Longhorn Beetle Desmocerus californicus dimorphus and Several Congeners (Cerambycidae: Lepturinae)
Source: PLoS One. 2014 Dec 18;9(12):e115498. doi: 10.1371/journal.pone.0115498 (PMC4270785; doi:10.1371/journal.pone.0115498)
Supplement: S1 Table — Location data and descriptions of habitat for the three conservation units within the Sacramento River National Wildlife Refuge where dose response studies were conducted for VELB. GPS coordinates indicate the position of the first trap in each replicate. (DOC) [file pone.0115498.s002.doc]

Table S1

| **Name of unit** | **GPS coordinates, Lat./Long.** | **Type of Habitat** |
| --- | --- | --- |
| McIntosh Landing South | 39.7685216 -122.0314689 | Riparian woodland – restored |
| McIntosh Landing South | 39.7687314 -122.0306811 | Riparian woodland - native |
| Ord Bend | 39.6270056 -121.0003232 | Riparian woodland – restored |
| Ord Bend | 39.6276696 -121.9964951 | Riparian woodland - restored |
| Pine Creek | 39.7447101 -121.9769735 | Riparian woodland – native |
| Pine Creek | 39.7396583 -121.9789903 | Elderberry savanna- native |
